# 

**Published:** 1897-12

**Authors:** 


					﻿Southern Medical Record.
A Monthly Journal of Medicine and Surgery.
Vol. XXVII. ATLANTA, GA., DECEMBER, 1897.	No. 12
BERNARD WOLFF, M. D., Editor.
Associate Editors :
A. W. GRIGGS, M. D.
WILLIS F. WESTMORELAND, M. D.
j. McFadden gaston; m. d.
E. Y. CLARKE, General Manager.
B. M. ZETTLER, Business Manager.
Entered at the Post-office at Atlanta, Ga., as Second-class Matter.
The Foote & Davies Co., Atlanta, Ga.
				

